# Ethnicity and the tumour characteristics of invasive breast cancer in over 116,500 women in England

**DOI:** 10.1038/s41416-021-01409-7

**Published:** 2021-05-26

**Authors:** Toral Gathani, Gillian Reeves, John Broggio, Isobel Barnes

**Affiliations:** 1 Cancer Epidemiology Unit, Nuffield Department of Population Health, University of Oxford, Oxford, UK; 2 Department of Breast Surgery, Oxford University Hospitals NHS Foundation Trust, Oxford, UK; 3 National Cancer Registration and Analysis Service, Public Health England, Birmingham, UK

**Keywords:** Breast cancer, Cancer epidemiology

## Abstract

**Background:**

Ethnic minority women are commonly reported to have more aggressive breast cancer than White women, but there is little contemporary national evidence available.

**Methods:**

We analysed data from the National Cancer Registration and Analysis Service on women diagnosed with invasive breast cancer during 2013–2018. Multivariable logistic regression yielded adjusted odds ratios (and 95% confidence intervals) of less favourable tumour characteristics (high stage, high grade, ER negative, Her2 positive) by ethnicity (black African, black Caribbean, Indian, Pakistani and white) in younger (30–46 years) and older (53–70 years) women.

**Results:**

In 24,022 women aged 30–46 at diagnosis, all ethnic minority groups apart from Indian women had a significantly greater odds of certain less favourable tumour characteristics compared to white women in fully adjusted models. In 92,555 women aged 53–70, all ethnic minorities had a significantly greater adjusted odds of several of the less favourable tumour characteristics. These differences were most marked in black African and black Caribbean women.

**Conclusions:**

Ethnic minority women are at greater risk of breast cancers with less favourable characteristics, even after allowing for age and other potential confounders. These differences are greater in older than younger women, and in the Black rather than South Asian ethnic groups.

## Background

Breast cancer is the second leading cause of cancer related death in women in England.^1^ Reliable national cancer mortality rates by ethnicity are not available as ethnicity is not routinely captured on death certificates,^2^ but survival rates have been reported to differ by ethnicity in some small studies.^3–9^ Poorer prognosis is associated with more advanced stage at diagnosis, higher grade, lack of oestrogen receptor expression (ER negative) and/or Herceptin receptor over-expression (Her2 positive).^7^ Observed variations in survival from breast cancer among ethnic groups may therefore be influenced by different patterns of the tumour characteristics of breast cancer.

The most recent population data available for ethnicity are from the 2011 census and show that 86% of the population in England and Wales is recorded as white. Among the remaining non-white population the single largest ethnic groups are Indian, Pakistani, black Caribbean and black African.^10^

Previous studies of tumour characteristics of breast cancer in relation to ethnicity have been limited by either small sample size, large amounts of missing data or crude ethnic groupings, which could mask differences between distinct groups. Such studies have also largely been confined to single ethnically dense regions of the country.^6,8,9,11–13^ Apparent variations in tumour characteristics in different ethnic groups may be due, at least in part, to differences in average age at diagnosis and by factors that influence health-seeking behaviour, such as deprivation, comorbidity and attendance for routine mammographic screening. These factors all vary by ethnicity^14–19^ and need to be taken account of when examining differences in tumour characteristics in these groups.

The National Cancer Registration and Analysis Service (NCRAS) is part of Public Health England and collates data on all people diagnosed with cancer in England.^20^ The need for better data collection to improve cancer outcomes was highlighted by the UK Department of Health in 2011.^21^ Part of the overall cancer strategy to improve outcomes was the mandatory implementation of the Cancer Outcomes and Services Dataset (COSD) to all NHS Trusts by January 1, 2013.^22^ COSD is the national data standard for reporting cancer in NHS England and provides detailed data including tumour characteristics.^23^ As a result of the mandatory implementation of COSD, the completeness of the recording of the tumour characteristics of interest has improved significantly.^24^ In parallel, the completeness of ethnicity recording in national datasets has also improved considerably driven by legislation to promote equality in all public sector bodies.^2^

The availability of large contemporary datasets allows for high quality breast cancer related research, with a focus on ethnicity, to be conducted at a national level. In this paper, we use a large national dataset from NCRAS to report the tumour characteristics of breast cancer in relation to ethnicity in over 116,500 women in England.

## Methods

All cancer registrations for invasive breast cancer (ICD-10 50) in women in England diagnosed between January 1, 2006 and December 31, 2018 were extracted from NCRAS. A detailed description of the data resource profile is provided elsewhere.^23,24^ The analyses presented are restricted to the time period January 1, 2013 to December 31, 2018, during which time COSD reporting has been mandatory and data are most likely to be complete.

Where ethnicity was recorded, women were assigned to one of the five largest ethnic groups according to the 2011 census:^10^ black African, black Caribbean, Indian, Pakistani or white.

Information on the tumour characteristics for breast cancer are all mandatory items reported in the Cancer Outcomes and Services Dataset (COSD).^25^ The main outcomes of interest were four tumour characteristics; TNM stage at diagnosis (I–IV), histological grade,^1–3^ ER status (positive or negative) and Her2 status (negative or positive). For the purposes of this analysis, stage and grade were classified into two groups representing a less favourable versus a more favourable prognosis i.e. high stage (locally advanced stage 3 and metastatic stage 4) versus low stage (early breast cancer stage 1 or 2) and high grade (grade 3) versus low grade (grade 1 or 2). The categories chosen for analysis reflect clinical utility and are widely used and understood in clinical settings.

Other variables included in the analyses for the purposes of adjustment included age at diagnosis (in ~3-year age bands) and region of diagnosis (nine regions, representing the regional teams of the English Cancer Registry). In addition, a comorbidity score was calculated from Hospital Episode Statistics data in the 18 months before the breast cancer diagnosis using the Charlson Index (no comorbidity or some comorbidity). There are 17 contributing morbidities in the Charlson Index, including conditions such as cardiovascular disease and respiratory disease, with their defined ICD-10 codes.^26^ Socioeconomic status was measured by the income domain of the index of multiple deprivation score (in quintiles). NCRAS is linked to National Health Service Breast Screening Programme (NHSBSP) records to provide information about screening attendance in those women who are eligible for routine population-based mammographic screening and therefore a variable was derived, which indicated whether a woman attended the last screening invitation prior to her breast cancer diagnosis (attended/did not attend).

Ethical approval for the study was obtained from the North East Tyne and Wear South Research Ethics Committee.

### Statistical analysis

The relationships between ethnicity and other patient characteristics and tumour characteristics of women in the five main ethnic groups were assessed in two separate age groups at diagnosis: younger women aged 30–46 years and older women aged 53–70 years based on the criteria identified by the national screening review in 2018.^27^ Prior to 2018, the NHS Breast Screening Programme invited women for routine screening by use of birth-year age, defined as the current year minus birth year. In 2018, the programme began to use birthday age. To determine if a woman belonged to the age groups of interest, her age at diagnosis was calculated based on the definition used by the NHSBSP at that time. Analyses were conducted separately within these two age groups because they differ in terms of their opportunity to attend for routine population-based screening for breast cancer.^28^ Women aged 30–46 years will not yet have had opportunity to attend for routine population-based screening. Women aged between 47 and 49 years may or may not have been offered screening as part of the AgeX trial^29^ and were therefore excluded. Women aged 50–52 years were also excluded as they may not have yet been invited for routine screening, as invitations are issued in batches and first screens are offered to women from the time they reach 50 years of age at some point during the 3-year screening cycle.^28^ By the time a woman has reached the age of 53 years, she should have had the opportunity to accept or decline a screening invitation and would be offered routine screening until the age of 70 years.

Logistic regression models were used to estimate odds ratios (ORs) and 95% confidence intervals separately for each of the four less favourable tumour characteristics; high stage, high grade, ER negative, Her2 positive versus the more favourable tumour characteristics; low stage, low grade, ER positive, Her2 positive, by ethnicity, within the two age groups. In both populations of women, analyses were initially adjusted for age, region and year of diagnosis. Adjustment was then made for factors that are likely to influence health-seeking behaviour such as deprivation and comorbidity, as well as history of screening attendance in the women aged 53–70.

Missing values for any of the adjustment variables in either model were assigned to a separate missing category for that variable. For each outcome, a sensitivity analysis was conducted, which was restricted to women with information available on all confounders. The reduction in the likelihood ratio X^2^ statistic associated with ethnicity in the model after adjustment of each variable was calculated, as a measure of the degree to which confounding by the adjustment variable would likely explain any observed association between ethnicity and the risk of the less favourable tumour characteristics.^30^

## Results

From the NCRAS data, 244,135 women were registered with a diagnosis of unilateral invasive breast cancer (ICD-10 C50) between January 1, 2013 and December 31, 2018. Among these women, 221,885 women (90.9%) had a recorded ethnicity in one of the five groups of interest. 24,022 (10.8%) of these women were aged 30–46 years of age and 92,555 (41.7%) of these women were aged 53–70 years at diagnosis and formed the two populations for analysis.

For the adjustment variables, data were complete for age at diagnosis, region, year of diagnosis and the measure of comorbidity using the Charlson Index. Information on deprivation was missing for less than 0.1% of the population, and a record of attendance at the last routine screen before cancer diagnosis was missing for 6.5% of women aged 53–70 years at diagnosis and ranged from 6.5 to 8.9% across the ethnic groups.

The characteristics of the younger women are summarised in Table 1. Of the 24,022 younger women aged 30–46 years at diagnosis, 91.6% were white; the remaining women were Indian (2.5%), black Caribbean (1.4%), Pakistani (2.0%) and black African (2.5%). The average age at diagnosis was similar and ranged from 39.9 years in black African and Pakistani women to 40.7 years in white and black Caribbean women. Highly significant differences were observed for deprivation scores (*p* < 0.0001) and comorbidity (*p* = 0.0007) between the ethnic minority groups with almost half of black Caribbean, Pakistani and black African women in the most deprived quintile, compared to less than a fifth of Indian and white women. Pakistani women had the poorest health of all the ethnic groups with a fifth recording at least one comorbidity.

**Table 1:** The characteristics of women aged 30–46 years at diagnosis with breast cancer between 2013 and 2018.

	White	Indian	Pakistani	Black Carribean	Black African	*P*-value
	(*n* = 22,001)	(*n* = 604)	(*n* = 483)	(*n* = 325)	(*n* = 609)	
Patient characteristics						
Mean age at diagnosis (SD)	40.7 (4.3)	40.3 (4.2)	39.9 (4.2)	40.7 (4.3)	39.9 (4.3)	<0.001
Most deprived quintile	15.9 (3 490)	17.3 (104)	48.1 (232)	41.5 (134)	41.1 (248)	<0.001
Comorbidity	14.3 (3 137)	12.3 (74)	19.0 (92)	16.6 (54)	10.5 (64)	0.001
Tumour characteristics						
Stage 3	17.6 (3579)	17.6 (97)	20.4 (89)	23.8 (72)	26.6 (147)	<0.001
Grade 3	47.6 (10,196)	48.9 (288)	60.4 (279)	52.6 (163)	57.4 (334)	<0.001
ER negative	23.0 (3813)	26.8 (109)	25.6 (89)	23.3 (52)	30.1 (122)	0.005
Her2 positive	21.5 (3767)	20.6 (93)	20.3 (78)	17.5 (44)	22.3 (98)	0.521

In general, all ethnic minority women presented with higher proportions of the less favourable tumour characteristics other than Her2-positive disease. For example, compared to white women, black African women had significantly higher proportions of high stage disease (26.6% versus 17.6%, *p* < 0.0001), high grade disease (57.4% versus 47.6%, *p* < 0.0001) and ER-negative disease (30.1% versus 23.0%, *p* = 0.005). The overall proportion of missing data was 7.8% for stage, 2.7% for grade, 25.1% for ER status and 20.8% for Her2 status, with ethnic minority women having higher proportions of missing data.

**Fig. 1:**
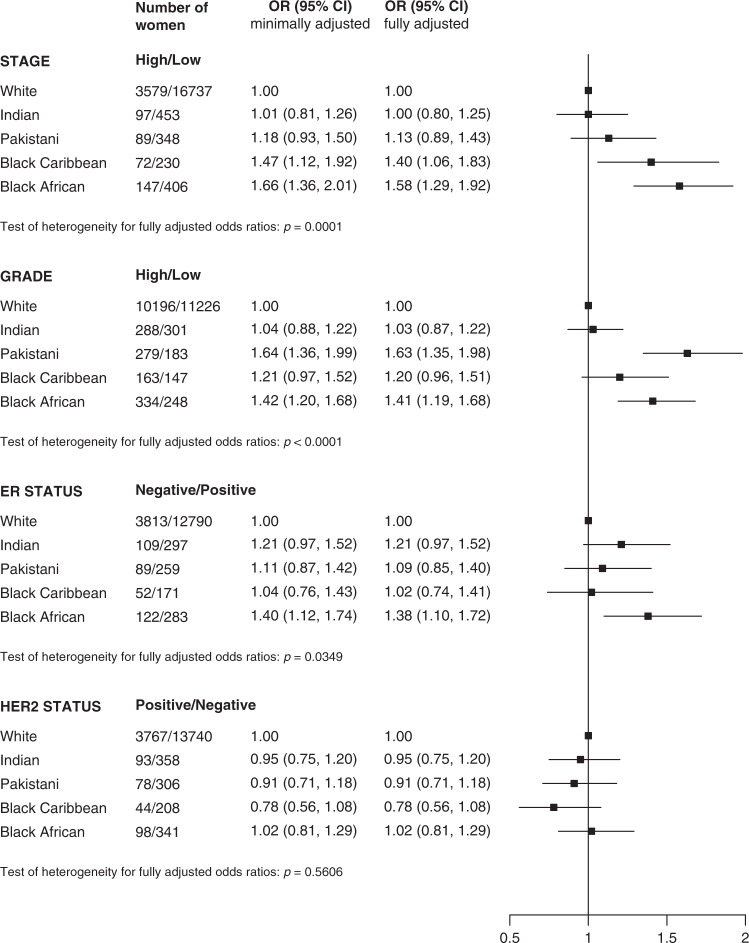
Odds ratio of high risk verus low risk tumour characteristics by ethnic group in women aged 30–46 years at diagnosis. OR = odds ratio. Minimally adjusted for age, region and year of diagnosis and then fully adjusted for deprivation and comorbidity.

The results of multivariate analysis of the odds of the less favourable tumour characteristics by ethnicity in the younger women are shown in Fig. 1. In analyses with minimal adjustment, Indian women had similar odds of all the less favourable tumour characteristics examined compared to white women. Black Caribbean women had significantly greater odds only for high stage disease and Pakistani women only for high grade disease, whereas Black African women had significantly greater odds for high stage, high grade and ER-negative disease compared to white women. Adjustment for measures of health-seeking behaviour resulted in attenuation of the risk of high stage disease to a degree, but greater odds of all the less favourable tumour characteristics remained in fully adjusted models e.g. black African women compared to white women in fully adjusted models; high stage disease OR 1.58 (95% CI 1.29–1.92), for high grade disease OR 1.40 (95% CI 1.18–1.66) and ER-negative disease OR 1.36 (95% CI 1.09–1.70).

The characteristics of the older women at diagnosis are shown in Table 2. Of the 92,555 women aged 53–70 years at diagnosis, 95.9% were white, 1.8% were Indian and <1% were Black Caribbean, Pakistani and black African, respectively. The average age at diagnosis ranged from 59.3 years in black Caribbean women to 61.8 years in white women. There were highly significant differences by ethnicity for deprivation (*p* < 0.0001) and the presence of at least one significant comorbidity (*p* < 0.0001). In general, all the ethnic minority women were more deprived compared to white Women, and in poorer health except for black African women. The overall attendance for the last screen before diagnosis where this was known, was highest for white, Indian and black Caribbean women (81.3–84.4%), and lower in black African women (75.7%) and Pakistani women (71.4%) (*p* < 0.001). The proportion of screen-detected cancers in women who had attended for screening was different by ethnicity. White, Indian and Pakistani women had similar proportions of screen-detected cancers (67.0–69.1%), but this proportion was lower in black African (61.7%) and black Caribbean women (59.4%) (*p* < 0.001).

**Table 2:** The characteristics of women aged 53–70 years at diagnosis with breast cancer between 2013 and 2018.

	White	Indian	Pakistani		Black Caribbean	Black African	*P*-value
	(*n* = 88,786)	(*n* = 1641)	(*n* = 786)		(*n* = 804)	(*n* = 538)	
Patient characteristics							
Mean age at diagnosis (SD)	61.8 (5.3)	61.2 (5.0)	60.7 (4.9)		59.3 (5.1)	59.5 (5.1)	<0.001
Most deprived quintile	14.4 (12,759)	18.9 (309)	46.6 (366)		35.1 (282)	41.7 (223)	<0.001
Comorbidity	25.9 (22,976)	38.1 (626)	55.6 (437)		34.7 (279)	27.5 (148)	<0.001
Attended for last screen	84.4 (70,126)	83.6 (1257)	71.4 (515)		81.3 (606)	75.7 (371)	<0.001
Tumour characteristics							
Stage 3	11.5 (9651)	13.2 (201)	15.3 (113)		17.3 (132)	22.5 (114)	<0.001
Grade 3	28.2 (24,317)	32.5 (513)	38.7 (292)		43.4 (334)	51.8 (270)	<0.001
ER negative	14.0 (9674)	19.8 (209)	19.5 (112)		26.2 (147)	34.0 (134)	<0.001
Her2 positive	13.7 (9788)	15.2 (186)	17.1 (109)		19.0 (111)	18.3 (75)	<0.001

There were significant differences by ethnicity in all the tumour characteristics examined (*p* < 0.0001 for all). Although in general, ethnic minority women had higher proportions of all the less favourable tumour characteristics compared to white women, these differences were more marked in black Caribbean and black African women compared to Indian and Pakistani women. The proportion of missing data in this age group was 5.4% for stage, 2.9% for grade, 22.3% for ER status and 20.0% for Her2 status, and again for ER and Her2 status the proportion of missing data were highest in ethnic minority women.

**Fig. 2:**
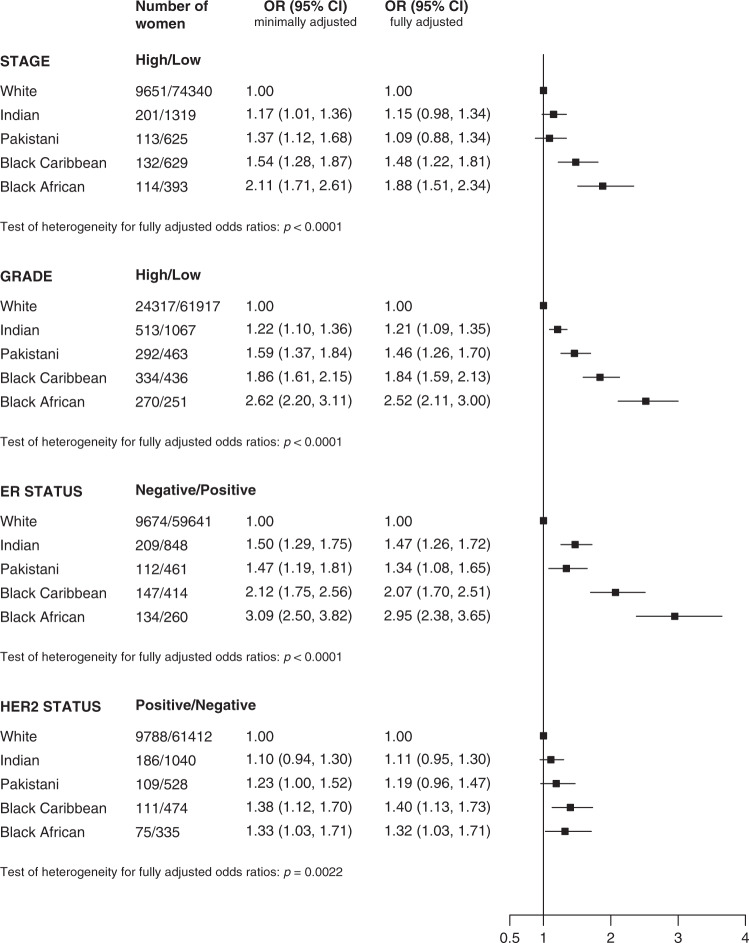
Odds ratio of high risk verus low risk tumour characteristics by ethnic group in women aged 53–70 years at diagnosis. OR = odds ratio. Minimally adjusted for age, region and year of diagnosis and then fully adjusted for deprivation, comorbidity and screening attendance.

The results for the multivariate analysis for each of the less favourable tumour characteristics by ethnicity in women aged 53–70 years are shown in Fig. 2. In minimally adjusted analyses, all ethnic minority women had significantly higher odds of high stage, high grade and ER-negative disease compared to white women. Although adjustment for confounders attenuated these risks to some degree, increased odds of high grade and ER-negative tumours were observed in all ethnic minority groups and increased odds of high stage and Her2 positive were observed in the two black subgroups, compared to white women. For example, black African women were around twice as likely to have high stage (OR 1.88 (95% CI 1.51–2.34)) and high grade (OR 2.42 (95% CI 2.03–2.89)) disease, and almost three times more likely to have ER-negative disease (OR 2.86 (95% CI 2.30–3.54)) compared with white women in fully adjusted models. The odds of Her2-positive disease were highest for black Caribbean women in fully adjusted models (OR 1.36 (95% CI 1.10–1.68)).

Restriction of analyses to women with information available on all confounders made little difference to the main findings. When the minimally adjusted associations with ethnicity were adjusted for each potential confounder, the likelihood ratio X^2^ statistics changed by less than 30% suggesting that residual confounding by comorbidity, deprivation and screening attendance does not account for the fully adjusted associations (Supplementary Tables 1 and 2).

## Discussion

In this large national study of contemporary data, clear differences were found in the tumour characteristics of breast cancer in women of different ethnic groups. Among younger women aged 30–46, and in the ethnic groups examined, only Indian women had a similar tumour characteristic profile compared to white women. For older women aged 53–70, all ethnic minority women had a less favourable tumour characteristic profile compared to white women, but these differences were more marked for black Africans and black Caribbeans. In general, differences in the risk of less favourable tumour characteristics were greater in the older than in the younger women.

These findings of higher risks of less favourable tumour characteristics in women of ethnic minority backgrounds, particularly black women, have been reported previously^6,8,9,11–13^ but to our knowledge, this is the first national study that has looked in detail at these associations in the five largest ethnic groups in England using contemporary data. Our findings are also consistent with other international studies located in more developed countries that have also reported more aggressive tumour characteristic profiles in their ethnic minority populations.^31,32^ However, limited robust comparisons between ethnic minority populations in different countries can be made as the routes and timeline of migration into these countries and access to healthcare systems are different. Our study is also the first national study to take into account factors that may influence tumour characteristics at diagnosis, and which are also likely to vary by ethnicity, including age at diagnosis, measures that influence health-seeking behaviour, such as deprivation, comorbidity and attendance for routine mammographic screening.^14–19^

The approach taken here to analyse the population in two distinct age groups also takes account of the different routes to diagnosis for breast cancer in different age groups. Younger women have breast cancer diagnosed largely as a result of presenting with a symptom and are known to present with higher rates of less favourable tumour characteristics.^33^ Whereas, older women could have the disease diagnosed as a result of presenting with a symptom or through asymptomatic detection through population-based screening. The proportion of ethnic minority women was higher among the younger women, which is a reflection of their generally younger age in the population,^10^ but the average age at breast cancer diagnosis in the two groups was similar for all ethnic groups.

The higher levels of deprivation and poorer health observed in the ethnic minority groups, and their potential subsequent effect on health-seeking behaviour for breast cancer are well known^2,3,6,17,18,34^ as are differences in uptake of screening, and these data provide further evidence for these differences. Although nationally, attendance for screening is reported at around 70%, in this study of women with breast cancer, attendance for the last screen before diagnosis is understandably higher. Interestingly, Indian and black Caribbean women attended at similar rates to white Women, and Pakistani and black African women were less likely to have attended their last screen prior to diagnosis and these findings are consistent with other studies. Attendance for screening in ethnic minority groups, is not only known to be influenced by factors including community values and beliefs,^35^ but also time since migration to the host country and the effect of acculturation. In black communities in the UK, first generation black African women are less likely to attend for screening than second generation black Caribbean women.^36^ In South Asian communities, lower uptake of screening are reported in Muslim compared to Hindu communities, which would be largely represented by Pakistanis and Indians respectively.^37^

In both younger and older women, Indian and Pakistani women had similar risks of high stage disease compared to white women, following adjustment of the confounders of deprivation, presence of comorbidity and attendance for screening. In comparison, black Caribbean and black African women in both age groups had higher risks of high stage disease at presentation even after adjustment for measures of health-seeking behaviour. As expected, the younger women in general had higher proportions of the less favourable tumour characteristics such as high grade and ER-negative disease compared to older women. Black Caribbeans and black Africans also had higher risks of Her2-positive disease.

Tumours with less favourable characteristics are more likely to be diagnosed in the interval between screens than at screening, and this could explain the lower proportion of screen-detected cancers observed in Black Caribbeans and black African women compared to the other groups in the older women.^38,39^ Adjustment by screening attendance made little difference to the risks of these tumour characteristics, suggesting that there are intrinsic differences in these tumour characteristic profiles in these older women.

It is unclear as to why there are differences in tumour characteristics between ethnic groups, and why these differences should be more marked in older women. The observed differences could be due, in part, to the personal characteristics of women that are known to influence the tumour characteristics of breast cancer. For example, ER-positive cancers are known to be associated with established risk factors for breast cancer such as parity and breast feeding,^40,41^ body mass index^42^ and use of hormone replacement therapy.^43^ Data from a large prospective study in the UK has shown that these factors vary materially by ethnicity^44^ and some of the increased risk of particularly ER-negative disease observed could be explained by these factors.

The main strengths of this paper are the use of a very large national dataset using routinely collected contemporary data, with high levels of recording of ethnicity (>95%), and significantly improved cancer registration data following the implementation of COSD. These data are now almost complete for stage and grade, and although, the overall completeness of ER and Her2 status remains lower, this dataset represents the most reliable national data that are available.^24^ The completeness of the ethnicity recording allows for detailed analysis by different ethnic groups and present findings in distinct groups of individuals, such as black Africans and black Caribbeans rather than just Blacks, and in Indians and Pakistanis, rather than just Asians.

Using routinely collected data have limitations, as little information is available on the personal characteristics of the women diagnosed with breast cancer, which can influence the profile of tumour characteristics as outlined above.

In conclusion, there are differences in the tumour characteristics of breast cancer in women of different ethnic minorities. Where these differences exist in comparison to white women, they are more marked in older than younger women, and in black Caribbean and black African than in Indian and Pakistani women. Further work is needed to understand what the drivers of these differences may be, and where these differences may impact outcomes and experience of the disease in different ethnic groups.

## Supplementary information

Supplementary Tables 1 and 2 Odds ratios for risks of less favourable versus more favourable tumour characteristics of breast cancer

## Data Availability

The data used in this study are held by University of Oxford under a data sharing contract with Public Health England and are not available for sharing. These data are available by individual application to the Office of Data Release at Public Health England (https://www.gov.uk/government/publications/accessing-public-health-england-data).

## References

[1] Cancer Research UK. *Breast cancer statistics*. https://www.cancerresearchuk.org/health-professional/cancer-statistics/statistics-by-cancer-type/breast-cancer (2020).

[2] Public Health England. *Local action on health inequalities: understanding and reducing ethnic inequalities in health*. https://www.gov.uk/government/publications/health-inequalities-reducing-ethnic-inequalities (2018).

[3] Maringe C, Li R, Mangtani P, Coleman MP, Rachet B (2015). Cancer survival differences between South Asians and non-South Asians of England in 1986–2004, accounting for age at diagnosis and deprivation. Br. J. Cancer.

[4] Farooq S, Coleman MP (2005). Breast cancer survival in South Asian women in England and Wales. J. Epidemiol. Community Health.

[5] Velikova G, Booth L, Johnston C, Forman D, Selby P (2004). Breast cancer outcomes in South Asian population of West Yorkshire. Br. J. Cancer.

[6] Morris M, Woods LM, Rogers N, O’Sullivan E, Kearins O, Rachet B (2015). Ethnicity, deprivation and screening: survival from breast cancer among screening-eligible women in the West Midlands diagnosed from 1989 to 2011. Br. J. Cancer.

[7] Moller H, Henson K, Luchtenborg M, Broggio J, Charman J, Coupland VH (2016). Short-term breast cancer survival in relation to ethnicity, stage, grade and receptor status: national cohort study in England. Br. J. Cancer.

[8] dos Santos Silva I, Mangtani P, de Stavola BL, Bell J, Quinn M, Mayer D (2003). Survival from breast cancer among South Asian and non-South Asian women resident in South East England. Br. J. Cancer.

[9] Copson E, Maishman T, Gerty S, Eccles B, Stanton L, Cutress RI (2014). Ethnicity and outcome of young breast cancer patients in the United Kingdom: the POSH study. Br. J. Cancer.

[10] Office for National Statistics. *2011 Census: Key Statistics for local authorities in England and Wales Table KS201EW: Ethnic group, local authorities in England and Wales*. https://www.ons.gov.uk/peoplepopulationandcommunity/populationandmigration/populationestimates/datasets/2011censuskeystatisticsforlocalauthoritiesinenglandandwales (2013).

[11] Jack RH, Davies EA, Moller H (2009). Breast cancer incidence, stage, treatment and survival in ethnic groups in South East England. Br. J. Cancer.

[12] Bowen R, Duffy SW, Ryan DA, Hart IR, Jones JL (2008). Early onset fo breast cancer in a group of British black women. Br. J. Cancer.

[13] Jack RH, Davies EA, Renshaw C, Tutt A, Grocock MJ, Coupland VH (2013). Differences in breast cancer hormone receptor status in ethnic groups: a London population. Eur. J. Cancer.

[14] Gathani, T., Chiuri, K., Broggio, J., Reeves, G. & Barnes, I. Ethnicity and the surgical management of early invasive breast cancer in over 164 000 women. *Br. J. Surg*. 10.1002/bjs.11865 (2020). [Epub ahead of print]. 10.1002/bjs.11865 PMC8210682 34043777

[15] Moser K, Patnick J, Beral V (2009). Inequalities in reported use of breast and cervical screening in Great Britain: analysis of cross sectional survey data. BMJ.

[16] Jack RH, Møller H, Robson T, Davies EA (2014). Breast cancer screening uptake among women from different ethnic groups in London: a population-based cohort study. BMJ Open.

[17] Joseph Rowntree Foundation University of Manchester. *Ethnicity and deprivation in England: How likely are ethnic minorities to live in deprived neighbourhoods?* https://hummedia.manchester.ac.uk/institutes/code/briefingsupdated/ethnicity-and-deprivation-in-england-how-likely-are-ethnic-minorities-to-live-in-deprived-neighbourhoods%20(1).pdf (2013).

[18] Joseph Rowntree Foundation University of Manchester. *Which ethnic groups have the poorest health? Ethnic health inequalities 1991 to 2011*. http://hummediamanchester.ac.uk/institutes/code/briefingsupdated/which-ethnic-groups-have-the-poorest-healthpdf (2013).

[19] Massat NJ, Douglas E, Waller J, Wardle J, Duffy SW (2015). Variation in cervical and breast cancer screening coverage in England: a cross-sectional analysis to characterise districts with atypical behaviour. BMJ Open.

[20] Public Health England. *National Cancer Registration and Analysis Service (NCRAS)*. https://www.gov.uk/guidance/national-cancer-registration-and-analysis-service-ncras (2019).

[21] Department of Health. *Improving Outcomes: A Strategy for Cancer*. https://www.gov.uk/government/publications/the-national-cancer-strategy (2011).

[22] Richards, M. *Implementation of the Cancer Services and Outcomes Dataset Information Standard*. https://www.gov.uk/government/publications/implementing-the-cancer-outcomes-and-services-dataset-information-standard (2012).

[23] Public Health England. *The National Cancer Registration and Analysis Service: a guide to cancer data and working with us.* https://www.ndrs.nhs.uk/wp-content/uploads/2020/07/National_Cancer_Registration_Guide-1pdf (2020).

[24] Henson KE, Elliss-Brookes L, Coupland VH, Payne E, Vernon S, Rous B (2020). Data resource profile: National Cancer Registration Dataset in England. Int. J. Epidemiol..

[25] National Cancer Intelligence Network. *Cancer Outcomes and Services Dataset (COSD)*. http://www.ncin.org.uk/collecting_and_using_data/data_collection/cosd (2020).

[26] Armitage JN, van der Meulen JH, on behalf of the Royal College of Surgeons Co-morbidity Consensus Group. (2010). Identifying co-morbidity in surgical patients using administrative data with the Royal College of Surgeons Charlson Score. Br. J. Surg..

[27] Richards, M. *The Independent Breast Screening Review 2018*. https://www.gov.uk/government/publications/independent-breast-screening-review-report (2018).

[28] National Health Service Breast Screening Programme. *When it’s offered—Breast cancer screening*. https://www.nhs.uk/conditions/breast-cancer-screening/when-its-offered/ (2019).

[29] Moser K, Sellars S, Wheaton M, Cooke J, Duncan A, Maxwell A (2011). Extending the age range for breast screening in England: pilot study to assess the feasibility and acceptability of randomization. J. Med. Screen.

[30] Parish S, Peto R, Palmer A, Clarke R, Lewington S, Offer A (2009). The joint effects of apolipoprotein B, apolipoprotein A1, LDL cholesterol, and HDL cholesterol on risk: 3510 cases of acute myocardial infarction and 9805 controls. Eur. Heart J..

[31] Brennan M (2017). Breast cancer in ethnic minority groups in developed nations: case studies of the United Kingdom and Australia. Maturitas.

[32] Chlebowski RT, Chen Z, Anderson GL, Rohan T, Aragaki A, Lane D (2005). Ethnicity and breast cancer: factors influencing differences in incidence and outcome. J. Natl Cancer Inst..

[33] Azim HA, Partridge AH (2014). Biology of breast cancer in young women. Breast Cancer Res..

[34] National Cancer Intelligence Network. *Breast Cancer: Deprivation—NCIN Data Briefing*. http://www.ncin.org.uk/publications/data_briefings/breast_cancer_deprivation (2013).

[35] Vrinten C, Wardle J, Marlow LA (2016). Cancer fear and fatalism among ethnic minority women in the United Kingdom. Br. J. Cancer.

[36] Jones CE, Maben J, Lucas G, Davies EA, Jack RH, Ream E (2015). Barriers to early diagnosis of symptomatic breast cancer: a qualitative study of Black African, Black Caribbean and White British women living in the UK. BMJ Open.

[37] Szczepura A, Price C, Gumber A (2008). Breast and bowel cancer screening uptake patterns over 15 years for UK south Asian ethnic minority populations, corrected for differences in socio-demographic characteristics. BMC Public Health.

[38] Niraula S, Biswanger N, Hu P, Lambert P, Decker K (2020). Incidence, characteristics, and outcomes of interval breast cancers compared with screening-detected breast cancers. JAMA Netw. Open.

[39] Blanks RG, Wallis MG, Alison RJ, Given-Wilson RM (2021). An analysis of screen-detected invasive cancers by grade in the English breast cancer screening programme: are we failing to detect sufficient small grade 3 cancers?. Eur. Radiol.

[40] Ritte R, Tikk K, Lukanova A, Tjønneland A, Olsen A, Overvad K (2013). Reproductive factors and risk of hormone receptor positive and negative breast cancer: a cohort study. BMC Cancer.

[41] Lambertini M, Santoro L, Del Mastro L, Nguyen B, Livraghi L, Ugolini D (2016). Reproductive behaviors and risk of developing breast cancer according to tumor subtype: a systematic review and meta-analysis of epidemiological studies. Cancer Treat. Rev..

[42] van den Brandt PA, Ziegler RG, Wang M, Hou T, Li R, Adami HO (2021). Body size and weight change over adulthood and risk of breast cancer by menopausal and hormone receptor status: a pooled analysis of 20 prospective cohort studies. Eur. J. Epidemiol..

[43] Collaborative Group on Hormonal Factors in Breast Cancer. (2019). Type and timing of menopausal hormone therapy and breast cancer risk: individual participant meta-analysis of the worldwide epidemiological evidence. Lancet.

[44] Gathani T, Ali R, Balkwill A, Green J, Reeves G, Beral V (2013). Ethnic differences in breast cancer incidence in England are due to differences in known risk factors for the disease: prospective study. Br. J. Cancer.

